# Hyperkinetic manifestations in sporadic parkinsonism: An exploratory case series with APOE and NOS3 variants

**DOI:** 10.1016/j.prdoa.2026.100474

**Published:** 2026-06-19

**Authors:** Sara Varanese, Rossella Ferrante, Maria Pia Buongarzone, Stefano Viola, Luisa Fiorelli, Daniela Travaglini, Paola Ajdinaj, Rino Speranza, Laura Bonanni

**Affiliations:** a Neurology Clinic, San Pio Hospital Via de Lellis, 66054 Vasto, Italy; b CAST - Center for Advanced Studies and Technology, Via Luigi Polacchi 11, 66100 Chieti, Italy; c Department of Medicine and Aging Sciences, University G. d'Annunzio of Chieti-Pescara, Italy

**Keywords:** Parkinsonism, Chorea, Tauopathy, APOE, NOS3

## Abstract

Background Hyperkinetic motor features are uncommon in Parkinson's disease and related parkinsonisms and may reflect underlying genetic susceptibility. Methods We describe three sporadic patients with parkinsonism and prominent hyperkinetic manifestations who underwent clinical exome sequencing. To provide preliminary context for these observations, we reviewed genetic data from an additional cohort of 11 patients with sporadic parkinsonism who underwent clinical exome sequencing in our center but did not exhibit early hyperkinetic manifestations Results All patients carried heterozygous variants in genes involved in vascular, inflammatory, and tau related pathways (NOS3 c.774 T > C; APOE c.388 T > C). Clinical phenotypes ranged from late-onset parkinsonism with severe choreic dyskinesias to young-onset dystonic parkinsonism with excellent dopaminergic responsiveness. Dopaminergic dysfunction was supported by functional neuroimaging. Conclusions These cases suggest that APOE and NOS3 variants may act as susceptibility modifiers, predisposing to hyperkinetic phenotypes within the parkinsonian spectrum.

## 1 Introduction

The coexistence of hyperkinetic movements in the context of parkinsonism represents a diagnostic and pathophysiological challenge. Chorea, dystonia, and excessive dyskinesias may arise from dopaminergic treatment, autoimmune or metabolic disorders, or underlying genetic susceptibility. Increasing evidence suggests that common genetic variants involved in tau metabolism, neuroinflammation, and vascular integrity may modulate clinical phenotypes in sporadic parkinsonian disorders.

Among these, APOE and NOS3 variants have been implicated in neurodegenerative processes through mechanisms involving oxidative stress, endothelial dysfunction, blood–brain barrier disruption, and tau hyperphosphorylation [1], [2], [3], [4].

However, their contribution to hyperkinetic manifestations within parkinsonism remains poorly explored. Here, we report three cases of sporadic parkinsonism with prominent hyperkinetic features in whom heterozygous APOE or NOS3 variants were identified. These observations are presented as exploratory and hypothesis-generating, with the aim of highlighting potential sources of phenotypic variability rather than implying a causal or associative relationship.

## 2 Case descriptions

### 2.1 Case 1

**Sex/Age/education:** Male, 73 years-old, 13 years of formal education (high school diploma).

#### 2.1.1 History and clinical presentation

The patient was diagnosed with rigid-type Parkinson's disease in 2017. He had no family history of neurodegenerative disorders. Initial treatment with pramipexole was titrated up to 1.05 mg/day. L-dopa (300 mg/day) was added in 2018. In 2019, opicapone was introduced to manage ON–OFF fluctuations but was discontinued after two weeks due to severe dyskinesias involving the head, trunk, and upper limbs. Safinamide 50 mg/day was subsequently attempted, leading to further worsening of dyskinesias and disabling freezing of gait. Cognitive function remained preserved (MoCA score = 26/30), with no evidence of executive dysfunction and only a mild deficit in delayed recall of previously learned verbal material. No evidence of RBD, hallucinations or autonomic dysfunction.

#### 2.1.2 Investigations

Brain MRI was unremarkable. DAT-SPECT demonstrated reduced tracer uptake in the putamina, more pronounced on the left side. Metabolic screening was negative. Genetic testing identified heterozygosity for two variants in the NOS3 gene: the missense polymorphism NM_000603.5:c.894G > T (p.Glu298Asp; rs1799983) and the synonymous variant NM_000603.5:c.774 T > C (p.Asp258=).

The combination of these variants is consistent with the heterozygous NOS3 haplotype containing the 298Asp allele, which has been associated with altered endothelial nitric oxide synthase activity and potential modulation of vascular and dopaminergic pathways.

### 2.2 Treatment and evolution

Subcutaneous apomorphine infusion (2.5 mg/day) resulted in improvement of motor fluctuations and dyskinesias but was discontinued after three months due to severe skin reactions. Low-dose foslevodopa/foscarbidopa infusion (0.17 ml/h) reduced OFF time but induced continuous dyskinesias. The patient ultimately returned to fractionated oral L-dopa therapy (350 mg/day), achieving the best balance between symptom control and tolerability, although mild–moderate peak-dose dyskinesias and 3–4 OFF hours per day persisted. MoCA score in 2025 remained 26/30.

### 2.3 Case 2

**Sex/Age/education:** Female, 71 years-old, 8 years of formal education.

#### 2.3.1 History and clinical presentation

The patient had no family history of neurodegenerative disease. At age 67, she developed progressive choreic movements of the face and head, later involving the upper limbs. Over time, she exhibited global bradykinesia, rigidity (predominantly in the lower limbs), apathy, and obsessive–compulsive symptoms. Levodopa therapy improved parkinsonian features but mildly exacerbated dyskinesias. Cognitive evaluation revealed mild impairment (MoCA = 20/30), with predominant executive dysfunction, including deficits in attention and cognitive flexibility. No evidence of RBD, hallucinations or autonomic dysfunction.

Based on available clinical features, the case was best classified as parkinsonism with prominent choreic features of uncertain etiology, not fulfilling criteria for a specific parkinsonian syndrome according to current MDS criteria.

#### 2.3.2 Investigations

Brain MRI was normal. DAT-SPECT and FDG-PET could not be completed due to continuous involuntary movements. Genetic testing revealed heterozygosity for *APOE* c.388 T > C.

#### 2.3.3 Treatment and evolution

Clozapine 50 mg/day was initiated for behavioral symptoms, leading to an unexpected and marked improvement of choreic movements, although mild dyskinesias persisted. At two-year follow-up, choreic movements were mild and intermittent, with a MoCA score of 19/30.

### 2.4 Case 3

**Sex/Age/education:** Female, 23 years-old, 18 years of formal education (University degree).

#### 2.4.1 History and clinical presentation

The patient had no family history of movement disorders. At age 22, she developed cervical dystonia associated with rigidity and bradykinesia of the left upper limb. Symptoms progressed over approximately one year, resulting in functional impairment. No RBD, cognitive, psychiatric, or autonomic symptoms were detected. The clinical picture is consistent with young-onset parkinsonism with dystonia, within the spectrum of parkinsonian disorders according to current MDS criteria.

#### 2.4.2 Investigations

Brain MRI was unremarkable. DAT-SPECT showed reduced dopaminergic uptake in the right putamen, consistent with presynaptic nigrostriatal dysfunction. Laboratory screening was negative.

Genetic testing identified heterozygosity for the APOE variant NM_000041.4:c.388 T > C (p.Cys130Arg; rs429358).

This variant, together with c.526C > T (p.Arg176Cys; rs7412), defines the classical APOE haplotypes (ε2, ε3, ε4). In the absence of the c.526C > T variant, the detected genotype is consistent with the ε4 allele in heterozygous state (ε3/ε4 haplotype).

#### 2.4.3 Treatment and evolution

Levodopa/benserazide was titrated to 300 mg/day, resulting in marked and sustained improvement of rigidity and bradykinesia. Cervical dystonia was effectively treated with botulinum toxin injections (onabotulinumtoxinA, 100 IU), with significant reduction of abnormal posturing and pain.

### 2.5 Additional cohort analysis

To explore whether the identified variants could be associated with a specific clinical phenotype, genetic data from an additional cohort of 11 patients with sporadic parkinsonism who had undergone clinical exome sequencing at the same center were reviewed. These patients did not present early or prominent hyperkinetic manifestations and had no known pathogenic variants associated with monogenic parkinsonism.

## 3 Methods

Clinical Exome Sequencing (CES) was performed using the Illumina NextSeq 500Dx platform with the ClinEx Pro capture kit. Bioinformatic analysis included alignment to the hg19 reference genome and variant annotation using Geneyx software. Clinical evaluations comprised standardized neurological and neuropsychological assessments, laboratory screening, and neuroimaging. Clinical diagnoses were assigned according to current Movement Disorder Society (MDS) clinical diagnostic criteria [5], where applicable.

### 3.1 Genetic variant interpretation

Variants were interpreted according to the American College of Medical Genetics and Genomics (ACMG) guidelines [6]. Filtering criteria included minor allele frequency thresholds, predicted functional impact, and known disease associations. Particular attention was given to the distinction between rare potentially pathogenic variants and common polymorphisms.

## 4 Discussion

This case series describes three patients with sporadic parkinsonism presenting with prominent hyperkinetic manifestations in whom heterozygous APOE or NOS3 variants were identified. The clinical presentations were heterogeneous, including treatment-sensitive dyskinesias, choreic-parkinsonian syndrome, and dystonic parkinsonism, and do not define a unified clinico-genetic entity. Rather, these cases are presented to illustrate the occurrence of marked hyperkinetic features across different parkinsonian phenotypes.

To provide preliminary context, we reviewed genetic data from an additional cohort of 11 patients with sporadic parkinsonism evaluated at our center who did not exhibit early or prominent hyperkinetic manifestations. None carried the APOE or NOS3 variants identified in the present cases. Given the very small sample size and lack of statistical power, this observation should be interpreted with extreme caution and cannot be considered evidence of enrichment or association.

Variants were interpreted according to the guidelines of the American College of Medical Genetics and Genomics (ACMG). The selection criteria included thresholds for minor allele frequency, predicted functional impact, and known disease associations. Particular attention was paid to distinguishing between rare, potentially pathogenic variants and common polymorphisms. The genetic variants identified in this study are common polymorphisms in the general population and cannot be considered disease-causing. According to current variant interpretation frameworks, including ACMG criteria, these variants are classified as benign or likely benign and are not clinically actionable in the context of monogenic Parkinsonian disorders (PMID: 25741868). The APOE rs429358 variant contributes to the definition of the ε4 allele, a well-established genetic risk factor for neurodegenerative diseases (PMID: 28459480, 36167654). However, APOE ε4 is not considered causative and is neither necessary nor sufficient to determine disease onset. Similarly, the NOS3 p.Glu298Asp variant has been reported to be associated with variability in endothelial nitric oxide synthase activity, although its biological and clinical significance remains uncertain (PMID 22735951). Both variants are relatively frequent in the general population (APOE ε4 allele frequency approximately 10–20%; NOS3 rs1799983 minor allele frequency approximately 30%), further supporting their non-pathogenic nature. Their identification in the present cases is therefore most likely incidental. At most, these variants could be hypothesized to act as potential modifiers of disease expression, possibly contributing to inter-individual variability through mechanisms such as neuroinflammation, vascular dysfunction, or alterations in blood–brain barrier integrity (PMID 34635885). However, this remains speculative and unsupported by direct evidence in the present study. The absence of these variants in the small comparison cohort precludes any inference regarding enrichment or association, given both the limited sample size and the high population frequency of the polymorphisms analyzed. Overall, the genetic findings presented here do not support a causal or associative role of APOE or NOS3 variants in hyperkinetic manifestations within Parkinsonism. Rather, they highlight the importance of cautious interpretation of common genetic variants identified through exome sequencing, particularly in the absence of functional validation, segregation data, or robust statistical evidence.

Case-specific factors further limit mechanistic interpretation. In Case 1, hyperkinetic manifestations were strongly influenced by dopaminergic treatment adjustments, suggesting increased treatment sensitivity rather than an intrinsic hyperkinetic phenotype. However, it is noteworthy that dyskinesias occurred relatively early and with marked severity, which is not typically observed in the natural history of Parkinson's disease, and may reflect an individual susceptibility that cannot be fully explained by treatment factors alone. In Case 2, diagnostic confidence is limited by incomplete imaging data and a broad syndromic presentation. However, the combination of progressive parkinsonism with prominent choreic features and a consistent longitudinal clinical course supports the presence of an underlying neurodegenerative process, despite the inability to achieve a more precise nosological classification. In Case 3, young-onset dystonic parkinsonism with marked levodopa responsiveness raises a wide differential diagnosis, and although major causes were screened, alternative explanations cannot be fully excluded. Nevertheless, the clear evidence of presynaptic dopaminergic deficit and sustained levodopa responsiveness support the classification within a parkinsonian spectrum.

From a pathophysiological perspective, APOE and NOS3 have been implicated in processes such as neuroinflammation, vascular dysfunction, oxidative stress, and blood–brain barrier regulation. These mechanisms are biologically plausible contributors to phenotypic variability in neurodegenerative disorders. However, in the present study, no direct evidence of motor circuit hyperexcitability or specific mechanistic pathways was obtained, and such interpretations remain speculative.

A recent study by Conti et al. [7] reported distinct clinical and network-level features in patients with Parkinson's disease carrying APOE variants, suggesting a possible modulatory role. While these findings are of interest, they remain preliminary and cannot be directly extrapolated to the present cases.

Overall, our observations do not support a causal or associative relationship between APOE or NOS3 variants and hyperkinetic manifestations in parkinsonism. Rather, they highlight the need for larger, systematically characterized cohorts integrating genetic, clinical, and functional data to determine whether common genetic variants may contribute to phenotypic heterogeneity. At present, these findings should be considered exploratory and hypothesis-generating.

## CRediT authorship contribution statement

**Sara Varanese:** Writing – original draft, Investigation, Formal analysis, Data curation, Conceptualization. **Rossella Ferrante:** Writing – review & editing, Formal analysis, Data curation. **Maria Pia Buongarzone:** Investigation. **Stefano Viola:** Investigation. **Luisa Fiorelli:** Investigation. **Daniela Travaglini:** Investigation. **Paola Ajdinaj:** Investigation. **Rino Speranza:** Investigation. **Laura Bonanni:** Writing – review & editing, Investigation, Conceptualization.

## Ethical statement

Written informed consent was obtained from the patients involved in this paper.

## Funding statement

No funding to be declared.

## Declaration of competing interest

The authors declare that they have no known competing financial interests or personal relationships that could have appeared to influence the work reported in this paper.
